# Transcriptional Profiles of the Response of Methicillin-Resistant *Staphylococcus aureus* to Pentacyclic Triterpenoids

**DOI:** 10.1371/journal.pone.0056687

**Published:** 2013-02-20

**Authors:** Pooi Yin Chung, Lip Yong Chung, Parasakthi Navaratnam

**Affiliations:** 1 School of Medicine and Health Sciences, Monash University, Sunway Campus, Malaysia; 2 Department of Pharmacy, Faculty of Medicine, University of Malaya, Kuala Lumpur, Malaysia; The Roslin Institute, University of Edinburgh, United Kingdom

## Abstract

*Staphylococcus aureus* is an important human pathogen in both hospital and the community that has demonstrated resistance to all currently available antibiotics over the last two decades. Multidrug-resistant isolates of methicillin-resistant *S. aureus* (MRSA) exhibiting decreased susceptibilities to glycopeptides has also emerged, representing a crucial challenge for antimicrobial therapy and infection control. The availability of complete whole-genome nucleotide sequence data of various strains of *S*. *aureus* presents an opportunity to explore novel compounds and their targets to address the challenges presented by antimicrobial drug resistance in this organism. Study compounds α-amyrin [3β-hydroxy-urs-12-en-3-ol (AM)], betulinic acid [3β-hydroxy-20(29)-lupaene-28-oic acid (BA)] and betulinaldehyde [3β-hydroxy-20(29)-lupen-28-al (BE)] belong to pentacyclic triterpenoids and were reported to exhibit antimicrobial activities against bacteria and fungi, including *S*. *aureus*. The MIC values of these compounds against a reference strain of methicillin-resistant *S*. *aureus* (MRSA) (ATCC 43300) ranged from 64 µg/ml to 512 µg/ml. However, the response mechanisms of *S. aureus* to these compounds are still poorly understood. The transcription profile of reference strain of MRSA treated with sub-inhibitory concentrations of the three compounds was determined using Affymetrix GeneChips. The findings showed that these compounds regulate multiple desirable targets in cell division, two-component system, ABC transporters, fatty acid biosynthesis, peptidoglycan biosynthesis, aminoacyl-tRNA synthetase, ribosome and β-lactam resistance pathways which could be further explored in the development of therapeutic agents for the treatment of *S. aureus* infections.

## Introduction

Antibiotic resistance is a growing global threat to the effective treatment of infectious diseases. Over the past two decades resistance has been reported in many clinically important pathogens and to a broad range of antimicrobials in clinical use today [1], [2], [3]. This has been the driving force for the discovery of novel agents with antimicrobial activity that may potentially lead to therapeutic agents. The isolation of vancomycin, β-lactam, macrolide, tetracycline, aminoglycoside and quinolone-resistant strains of *S. aureus* provides a message that the availability of structurally novel antibacterials with multiple modes of action need to be part of the antibacterial armamentarium [4]. Over the past 30 years, only two new classes of antibiotics for the treatment of staphylococcal infections have been introduced: the oxazolidinones represented by linezolid, and the lipopeptide daptomycin [5]. However, reports of resistance to these antibiotics have also been reported [6]. Clearly, new approaches and innovative strategies are required to discover and identify agents that target essential bacterial targets in novel pathways, i.e. not targeted by currently used antibiotics, or novel targets in existing pathways. Promising targets for novel antibacterials against *S. aureus* include cell division, DNA replication and biosynthesis of fatty acid, peptidoglycan and protein [7].

To capture and exploit the wealth and diversity of genetic information of a variety of microorganisms, development of new technologies such as microarray provides a unique tool to understand the molecular basis of drug activity as it allows simultaneous analysis of the expression of all genes in the organism under any given condition. Transcriptional profiles generated by GeneChip analysis of bacteria have been used to investigate differential gene expression in response to antimicrobial agents [8], such as the response of *S. aureus* strain 8325-4 to antibiotics that act on cell wall such as oxacillin, D-cycloserine and bacitracin [9], and the effects of vancomycin on gene expression in *S. aureus* [10].

Plant-derived natural products have been reported to show anti-staphylococcal activities with micromolar MICs [11], [12], [13], [14], [15], [16]. In a preliminary study, the effects of 191 extracts towards different strains of bacteria were evaluated. Extracts from *Callicarpa farinosa* were shown to exhibit strong antimicrobial activities against MRSA [16]. Three pentacyclic triterpenoids, namely α-amyrin, betulinic acid and betulinaldehyde, have been investigated and found to have antibacterial activity against reference strains of methicillin-sensitive and methicillin-resistant *S. aureus*. Their activity was potentiated by currently used antistaphylococcal drugs [17]. In this study, the genome-wide transcription induced by the exposure of reference strain of *S. aureus* to pentacyclic triterpenoids was used to elucidate the mechanism of action of the compounds.

## Materials and Methods

### Bacterial Strains and Materials

The *S. aureus* strain used in this study was reference strain ATCC 43300, representing methicillin-resistant strains. The bacterial strain was maintained on Nutrient Agar (NA) (Difco, USA) slopes. Bacterial cultures used for microarray experiments were grown on cation-adjusted Mueller-Hinton broth (CAMHB II) (Difco, USA). Commercial sources of pentacyclic triterpenoids α-amyrin (AM) and betulinic acid (BA) were obtained from Sigma-Aldrich (USA), while betulinaldehyde (BE) was obtained from Advanced Technology and Industrial Co., Ltd (Hong Kong).

### Growth of *Staphylococcus aureus* in the Presence of AM, BA and BE

In order to obtain good microarray results, the concentrations of the compounds need to be optimized at sub-inhibitory concentrations that do not affect the growth of the organism [18], [19], [20], [21]. The treatment time with AM, BA and BE was determined by exposure of the organism to the study compounds and bacterial growth curve analysis. The bacterial culture was grown in CAMHB and incubated at 37°C with shaking at 200 rpm, to an optical density of 0.3 at 600 nm; i.e. at mid-logarithmic phase as culture conditions at this phase were most constant and RNA levels were at the highest due to high metabolic rate. The optical density of the cultures was measured using a spectrophotometer (Nanophotometer, Germany). The cultures were then distributed into 100 ml aliquots. AM, BA and BE were added to the aliquots to obtain concentrations of 1/2× MIC (32 µg/ml for AM and BA; 256 µg/ml for BE) and 1× MIC (64 µg/ml for AM and BA; 512 µg/ml for BE) [17]. The cultures were then incubated, and cell growth was spectrophotometrically monitored at OD 600 nm for 5 hours at time intervals of 30 minutes. Untreated bacterial culture was used as control. The growth curve for each treatment and concentration was carried out in triplicates.

### Total RNA Isolation and Purification

Three independent bacterial cultures for each treatment and control were prepared as biological replicates for total RNA isolation. After treatment of bacterial cells with the study compounds at concentrations and incubation times as determined from the growth curve, the cells were immediately stabilized with RNA Protect Bacteria Reagent (Qiagen, USA) to minimize RNA degradation before harvesting. Total RNA was isolated from treated and control bacterial cultures using the RNeasy Midikit (Qiagen) protocol. The cell wall of *S. aureus* was disrupted with Tris-EDTA (TE) buffer with 50 mg/ml lysozyme (Sigma Aldrich) containing proteinase K (Qiagen). DNA was removed using RNase-free DNase I (Qiagen). After purification, RNA concentration was determined by absorbance at 260 nm on Nanophotometer (1 absorbance unit is equivalent to 40 µg/ml RNA). RNA integrity was determined using Agilent RNA 6000 Nano chips (Agilent Technologies, USA). The procedure in preparing the assay, loading the chip and running the assay was carried out according to the manufacturer’s protocol at Malaysia Genome Institute, Bangi, Malaysia.

The total RNA analysis showed that the A_260_/A_280_ and A_260_/A_230_ readings of all the total RNA samples were in the range of 1.8 to 2.1 and a single peak at 260 nm indicating that the samples were free from contaminants such as proteins, phenol and salts, which could interfere with down-stream applications such as cDNA synthesis and microarray analysis. The RIN values were above 7.0, indicative of non-degraded RNA (supporting information Table S1).

### cDNA Synthesis, Fragmentation and Terminal Labeling

cDNA was synthesized with a starting material of 10 µg of the total RNA, according to manufacturer’s protocol (prokaryotic array, Affymetrix, USA). The procedure was carried out in a Mastercycler Gradient thermal cycler (Eppendorf, Germany). The cDNA obtained was then purified using a MinElute PCR Purification Column Kit (Qiagen) and quantified using Nanophotometer at 260 nm absorbance. A minimum of 1.5 µg cDNA was then fragmented in a reaction mix containing DNase I and DNase running buffer (Promega, USA). The fragmented cDNA was then applied directly to the terminal labeling reaction using GeneChip DNA Labeling Reagent (Affymetrix). The fragmented and labeled cDNA (target) was then used for hybridization.

### GeneChip Hybridization

The labeled fragmented cDNA from independent RNA preparations were hybridized onto a Genechip probe array masked with known genome of *S. aureus* N315 (methicillin-resistant strain), Mu50 (methicillin and vancomycin-resistant strain), COL (methicillin-resistant strain) and NCTC 8325 strains (methicillin-sensitive strain) (Affymetrix). The array contains probe sets of over 3300 *S. aureus* ORFs and over 4800 intergenic regions throughout the *S. aureus* genome. The labeled cDNA was hybridized, washed and scanned according to manufacturer’s protocol (prokaryotic array, Affymetrix). The data obtained was analyzed using the Affymetrix GeneChip Command Console software (AGCC).

### Microarray Analysis

For gene expression analysis, data from the Affymetrix platform were processed with GeneSpring version 11 software. Data was normalized to minimize systematic non-biological differences to reveal true biological differences. Significance analysis using ANOVA test with multiple testing correction Benjamini Hochberg to correct false discovery rate and asymptotic p-value computation was performed to determine the change of expression of a particular gene due to the effect of different treatment on *S. aureus*. In addition, fold-change analysis was also performed and reported as the up- or down-regulated fold change. To select the differentially expressed genes, threshold values of ≥2 fold-change between pentacyclic triterpenoid-treated samples and control samples and significance level of <5% were used. From the statistical analysis, the gene ontology (GO) analysis was carried out. The pathways affected by the differentially expressed genes were analyzed using the KEGG database (http://www.genome.jp/kegg/pathway.html). To group genes with similar expression profiles together, hierarchical clustering analysis was used in which an entity tree and a condition tree were generated.

### Validation of Gene Expression by Quantitative Real-time Quantitative PCR

To determine the validity of the microarray data, transcript level changes obtained with the microarray analysis were compared with those from quantitative real-time PCR (qPCR). Aliquots of cDNA preparations used in the microarray experiments were used for qPCR procedure. A list of genes and the primer sequences used for the real-time PCR analysis is shown in Table 1. The housekeeping gene *adhE* and *gyrA* were used as endogenous reference genes. The procedure was performed using the iCycler iQ5 Real Time PCR Detection System (Bio-Rad Laboratories, USA) with Quantitect SYBR Green PCR kit (Qiagen). For each gene, three biological replicates with three technical replicates each were used. Real-time cycler conditions were carried out according to manufacturer’s protocol: initial activation step at 95°C for 15 min, followed by 45 cycles of denaturation at 15 s and 94°C, annealing at 30 s and 53 to 60°C (as optimized for each target gene, data not shown), and extension at 30 s and 72°C. Melting curve analysis was also performed to verify PCR specificity. PCR efficiencies were derived from standard curve slopes in the iCycler software v3.1. Relative quantification based on the relative expression of the target gene compared to reference genes *adhE* and *gyrA* in treated and control samples was used to determine transcript level changes [22]. Paired student’s T test was performed to assess whether the means of a treated and control group (samples) is significantly different from each other using the SPSS software (PASW Statistic 18).

**Table 1:** Primers used in real-time PCR for validation of target genes.

Genesymbol	Sequence accession number	Amplicon length	Primer sequence	
			Forward	Reverse
ftsZ	1123860	85	AGCTGCAGAGGAATCTCGTGAACA	TCCGCCACCCATACCAGAAGTA
opp-1C	1125181	81	CGTGCGTGTGATGTTATGTTGGCA	TTCGGCACCCATTCCAAACAATGC
oppF	1122975	81	GGTGGGCAACGTCAGCGTGTAA	GGACACTGCCTCGTCGCAAACA
asnC	1124125	120	TCGGTGGATCTGAACGTGTGGA	GTGGCACACTACCATAACGACG
fabZ	1124802	131	TGAAGAAGGTCAACGTTGTGTGGC	CCGCACCTGTTTGAGCTAACGC
fmhB	1124980	151	AAGCGAGGTACGACAGTAGAACG	CATCTCCATCTTCATGCAACGCA
pbp2	1124121	90	CGTTTCTACGAACATGGCGCAC	GGCACCTTCAGAACCAAATCCACCA
ccrA	1122832	106	CAGCTTGGCCGAACTTGAATCAGA	ACCAAAGGGCGCATGGGTTG
mecR1	1122813	112	GTGCTCGTCTCCACGTTAATTCCA	GACTAACCGAAGAAGTCGTGTCAG
adhE	1122918	105	TTAGCGAAGTCGAACCGAACCCA	CTGAACCACCACCAAGTGCAATGA
gyrA	1122777	80	TATCCGCTTGTTGATGGCCAAGG	CGCGCTTCAGTATAACGCATTGC

## Results and Discussion

### Growth Profile of *Staphylococcus aureus* in the Presence of AM, BA and BE

**Figure 1:**
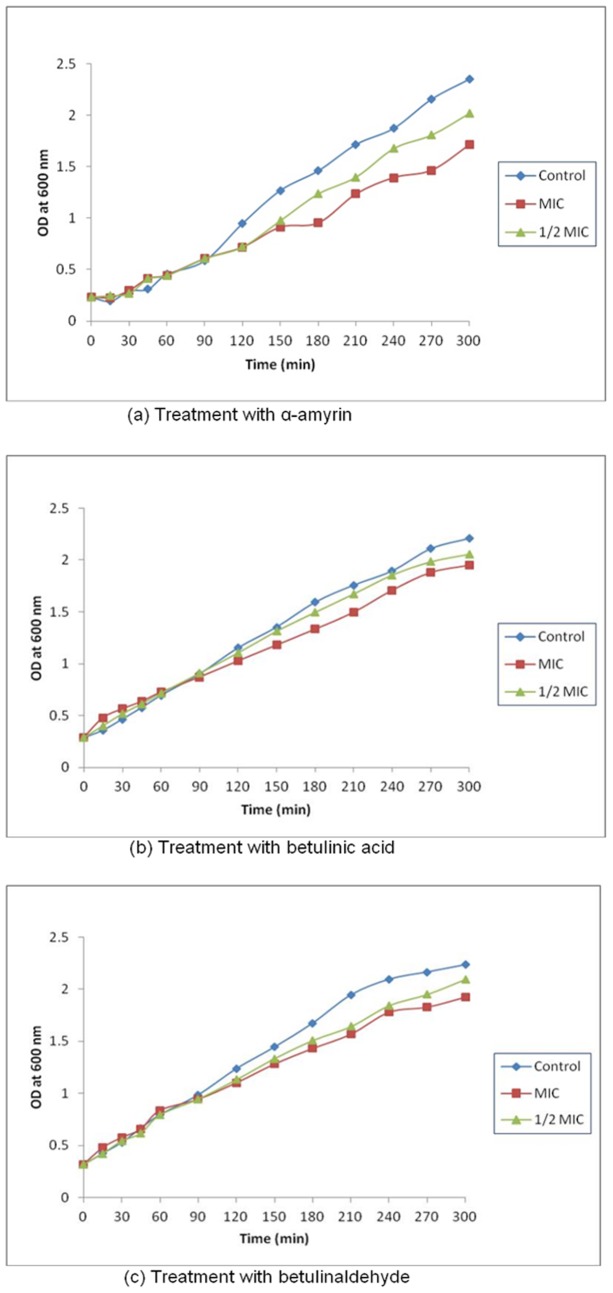
Growth curves of reference strain of methicillin-resistant *Staphylococcus aureus* treated with α-amyrin, betulinic acid and betulinaldehyde.

The effect of AM, BA and BE on the growth of reference strain of MRSA is as shown in Figure 1. The growth rate of the organism decreased at concentrations ½ MIC and MIC after 30 min for AM; and 45 min for BA and BE. After 300 min, the growth of the organism at ½ MIC was generally higher than the growth at MIC. These effects of the growth indicates that AM, BA and BE are bacteriostatic compounds as reported by Chung et al. [17]. Based on the growth curves, the organism was then treated with the three study compounds at ½ MIC (32 µg/ml for AM and BA; and 256 µg/ml for BE) and further incubated for 30 min (control and treatment with AM) and 45 min (treatment with BA and BE). Subinhibitory concentrations of the compounds were used in the microarray procedure to avoid secondary responses caused by high concentrations of the compounds.

### Overview of Transcriptional Profiles of *Staphylococcus aureus* Treated with Pentacyclic Triterpenoids

From the data processed using GeneSpring version 11 software, an enormous number of genes were significantly differentially regulated (p<0.05, fold change ≥2) in response to treatment with AM, BA and BE as compared to control (Table 2). For example, a total of 1265 genes were significantly affected in the treatment of reference methicillin-resistant strains with BE. There is also a vast number of the genes which are uncharacterized, without specific functional category and pathways, and with unknown function (Tables S2, S3).

**Table 2:** Total number of differentially regulated genes in response to α-amyrin, betulinic acid and betulinaldehyde.

Treatment	Up-regulated genes	Down-regulated genes	Total number of significantly regulated genes
α-amyrin	77	102	179
Betulinic acid	178	69	247
Betulinaldehyde	523	742	1265

Overall, the changes in expression levels of most genes were moderate with ratio in the range of 2- to 3-fold (Tables 3, 4, 5, 6). There are 31 genes coding for ribosome which were highly down-regulated (more than 5-fold and up to 21-fold), indicating that ribosome assembly may play a prominent role in the response of *S. aureus* to BE. Four genes in the fatty acid biosynthesis and three genes in the peptidoglycan biosynthesis were down-regulated more than 3-fold while three genes in the ABC transporters were at least 3-fold up-regulated after treatment with BE. ABC transporters and ribosomes are significantly regulated by all three pentacyclic triterpenoids. Only BE affects cell division, while only BA targets genes in β-lactam resistance and DNA replication pathways.

**Table 3:** Selected genes which are differentially up-regulated in response to α-amyrin, betulinic acid and betulinaldehyde.

N315 ORF	Gene symbol	Product	Fold change^δ^	Functional category
(A) Treatment with α-amyrin				
ABC transporters				
SA2253	opp-1C	Oligopeptide transporter permease	+2.1	Transport and binding proteins
(B) Treatment with betulinic acid				
ABC transporters				
SA2253	opp-1C	Oligopeptide transporter permease	+2.1	Transport and binding proteins
β-lactam resistance				
SA0039	mecR1	Methicillin resistance protein	+2.1	Transcription
DNA replication				
SA0057	ccrB	Cassette chromosome recombinase B	+2.2	DNA replication
SA0058	ccrA	Cassette chromosome recombinase A	+2.4	DNA replication
(C) Treatment with betulinaldehdye				
Two component system				
SAS066	agrD	AgrD protein	+2.1	Signal transduction mechanisms
Aminoacyl-tRNA biosynthesis				
SA0986	pheT	Phenylalanine-tRNA synthetase beta chain	+3.0	Translation
ABC transporters				
SA0109	sirC	Lipoprotein (iron complex transport protein)	+2.6	Inorganic ion transport
SA0110	sirB	Lipoprotein (iron complex transport protein)	+5.8	Inorganic ion transport
SA0198	oppF	Oligopeptide transport ATP-binding protein	+3.8	Inorganic ion transport
SA1214	opp2B	Oligopeptide transporter permease	+3.5	Inorganic ion transport

**Table 4:** Selected genes which are differentially down-regulated in response to α-amyrin.

N315 ORF	Gene symbol	Product	Fold change^δ^	Functional category
Two-component system				
SA0661	saeR	Response regulator	−3.9	Regulatory function
SA0060	saeS	Histidine protein kinase	−2.2	Regulatory function
SA1881	kdpA	Potassium-transporting ATPase subunit A	−2.6	Inorganic ion transport
SA1936	luxS	S-ribosylhomocysteinasae	−2.1	Signal transduction
Aminoacyl-tRNA biosynthesis				
SA0488	cysS	Cysteinyl-tRNA synthetase	−2.1	Translation
SA1106	proS	Prolyl-tRNA synthetase	−2.6	Translation
SA1287	asnC	Asparaginyl-tRNA synthetase	−2.4	Translation
SA1506	thrS	Threonyl-tRNA synthetase	−2.4	Translation
Fatty acid biosynthesis				
SA1073	fabD	Malonyl coA-acyl carrier protein transacylase	−2.5	Fatty acid and phospholipid metabolism
Peptidoglycan biosynthesis				
SA2057	fmhB	FmhB protein	−2.2	Cell envelope
Ribosome				
SAS042	rpmG	50s ribosomal protein L33	−2.0	Transcription
SA0354	rpsR	30s ribosomal protein S18	−2.1	Transcription
SA0497	rplJ	50s ribosomal protein L10	−2.5	Transcription

**Table 5:** Selected genes which are differentially down-regulated in response to betulinic acid.

N315 ORF	Gene symbol	Product	Fold change^δ^	Functional category
Two component system				
SA1883	kdpE	KDP operon transcriptional regulatory protein	−2.1	Signal transduction mechanisms
SA0068	kdpA	Potassium-transporting ATPase subunit A	−2.1	Signal transduction mechanisms
ABC transporters				
SA0198	oppF	Oligopeptide transport ATP-binding protein	−2.2	Inorganic ion transport
Ribosome				
SAS042	rpmG	50s ribosomal protein L33	−2.1	Transcription

**Table 6:** Selected genes which are differentially down-regulated in response to betulinaldehyde.

N315 ORF	Gene symbol	Product	Fold change^δ^	Functional category
Cellular process				
SA1029	ftsZ	Cell division protein FtsZ	−4.8	Cell division
SA1028	ftsA	Cell division protein FtsA	−5.2	Cell division
Two-component system				
SA1844	agrA	Accessory gene regulator A	−2.4	Signal transduction mechanisms
SA1881	kdpA	Potassium-transporting ATPase subunit A	−9.0	Signal transduction mechanisms
SA1883	kdpE	KDP operon transcriptional regulatory protein	−2.7	Signal transduction mechanisms
Aminoacyl-tRNA biosynthesis				
SA0209	serS	Seryl-tRNA synthetase	−2.7	Translation
SA0475	lysS	Lysyl-tRNA synthetase	−3.3	Translation
SA1106	proS	Propyl-tRNA synthase	−2.3	Translation
SA1287	asnC	Asparaginyl-tRNA synthase	−2.3	Translation
SA1506	thrS	Threonyl-tRNA synthase I	−2.6	Translation
Fatty acid biosynthesis				
SA0843	fabF	3-oxoacyl synthase	−3.4	Fatty acid and phospholipid metabolism
SA0889	fabI	Enoyl-(acyl carrier protein) reductase	−2.7	Fatty acid and phospholipid metabolism
SA1073	fabD	Malonyl coA-acyl carrier protein transacylase	−3.0	Fatty acid and phospholipid metabolism
SA1074	fabG	3-oxoacyl-{acyl-carrier protein} reductase	−3.9	Fatty acid and phospholipid metabolism
SA1901	fabZ	(3R)-hydroxymyristoyl ACP dehydratase	−3.7	Fatty acid and phospholipid metabolism
Peptidoglycan biosynthesis				
SA1251	murG	Undecaprenyldiphospho-muramoylpentapeptide beta-N-acetylglucosaminyltransferase	−3.9	Cell envelope
SA1283	pbp2	Penicillin binding protein 2	−4.5	Cell envelope
SA1561	murC	UDP-N-acetylmuramate L-alanine ligase	−2.5	Cell envelope
SA1902	murA	UDP-N-acetylglucoasmine 1-carboxyvinyltransferase	−5.1	Cell envelope
SA2057	fmhB	FmhB protein	−2.2	Cell envelope
Ribosome				
SA0503	rpsL	30S ribosomal protein S12	−7.3	Translation
SA2025	rpsM	30S ribosomal protein S13	−6.1	Translation
SA1081	rpsP	30S ribosomal protein S16	−2.9	Translation
SA2038	rpsQ	30S ribosomal protein S17	−10.2	Translation
SA0354	rpsR	30S ribosomal protein S18	−4.8	Translation
SA2043	rpsS	30S ribosomal protein S19	−4.8	Translation
SA1099	rpsB	30S ribosomal protein S2	−9.9	Translation
SA1414	rpsT	30S ribosomal protein S20	−6.6	Translation
SA1404	rpsU	30S ribosomal protein S21	−12.9	Translation
SAS052	rpsD	30S ribosomal protein S4	−5.7	Translation
SA0496	rplA	50S ribosomal protein L1	−6.1	Translation
SA2031	rpsE	30S ribosomal protein S5	−8.3	Translation
SA0352	rpsF	30S ribosomal protein S6	−5.7	Translation
SA0497	rplJ	50S ribosomal protein L10	−6.5	Translation
SA0495	rplK	50S ribosomal protein L11	−6.2	Translation
SA2017	rplM	50S ribosomal protein L13	−9.0	Translation
SA2037	rplN	50S ribosomal protein L14	−9.2	Translation
SA2029	rplO	50S ribosomal protein L15	−6.3	Translation
SA2040	rplP	50S ribosomal protein L16	−7.4	Translation
SA2022	rplQ	50S ribosomal protein L17	−3.7	Translation
SA2032	rplR	50S ribosomal protein L18	−7.6	Translation
SA1473	rplU	50S ribosomal protein L21	−6.9	Translation
SA2036	rplX	50S ribosomal protein L24	−9.7	Translation
SA0459	rplY	50S ribosomal protein L25	−5.4	Translation
SA1471	rpmA	50S ribosomal protein L27	−6.3	Translation
SA1067	rpmB	50S ribosomal protein L28	−6.3	Translation
SA2039	rpmC	50S ribosomal protein L29	−9.4	Translation
SA2047	rplC	50S ribosomal protein L3	−4.9	Translation
SA2030	rpmD	50S ribosomal protein L30	−6.2	Translation
SA1922	rpmE	50S ribosomal protein L31 type B	−4.8	Translation
SAS033	rpmF	50S ribosomal protein L32	−7.7	Translation
SAS042	rpmG	50S ribosomal protein L33 1,2,3	−8.4	Translation
SAS093	rpmH	50S ribosomal protein L34	−6.3	Translation
SA1503	rpmI	50S ribosomal protein L35	−6.0	Translation
SAS078	rpmJ	50S ribosomal protein L36	−21.0	Translation
SA2035	rplE	50S ribosomal protein L5	−6.3	Translation
SA0498	rplL	50S ribosomal protein L7/L12	−8.3	Translation
SA0014	rplI	50S ribosomal protein L9	−2.8	Translation

Differentially expressed genes in promising pathways such as cell division (*ftsZ*), ABC transporters (*opp-1C*), biosynthesis of fatty acid (*fabZ*), peptidoglycan (*fmhB*, *pbp2*) and protein (*asnC*), DNA replication (*ccrA*) and β-lactam resistance (*mecR1*) were selected to be validated by real-time PCR. The paired student’s T test analysis showed that all these selected genes were extremely statistically significantly different between bacterial cells treated with AM, BA or BE, and untreated cells. The difference in the expression of reference genes *adhE* and *gyrA* was considered not significantly different (Table S4).

A comparison of genes identified in the treatments of all three study compounds showed that genes *rpmG* and *kdpA*, which code for the protein of the large subunit 50S and potassium-transporting ATPase subunit A, respectively, are down-regulated. AM and BA showed up-regulation of *opp-1C* in the ABC transporter pathway, while *kdpE* in the two-component systems are similarly down-regulated in BA and BE. Treatment of the bacterial strain with AM and BE affected the highest number of similarly regulated genes, involving a total of seven genes in the two-component systems, fatty acid biosynthesis, peptidoglycan biosynthesis, aminoacyl-tRNA biosynthesis and ribosome pathways (Table 7). Generally, all three study compounds acted on more than one target/pathway that is unique to the microorganisms. This is a highly desirable property of an antibacterial agent and it could potentially delay the development of bacterial resistance to the compounds [23], [24].

**Table 7:** Similarly regulated genes in response to treatment with α-amyrin, betulinic acid and/or betulinaldehyde.

N315 ORF	Gene symbol	Product	Pathway
Treatment with α-amyrin, betulinic acid and betulinaldehyde			
SA1881	kdpA	Potassium-transporting ATPase subunit A	Two-component system
SAS042	rpmG	50s ribosomal protein L33 1,2,3	Ribosome
Treatment with α-amyrin and betulinic acid			
SA1881	kdpA	Potassium-transporting ATPase subunit A	Two-component system
SA2253	opp-1C	Oligopeptide transporter permease	ABC transporter
SAS042	rpmG	50s ribosomal protein L33 1,2,3	Ribosome
Treatment with α-amyrin and betulinaldehyde			
SA1881	kdpA	Potassium-transporting ATPase subunit A	Two-component system
SA1287	asnC	Asparaginyl-tRNA synthase	Aminoacyl-tRNA biosynthesis
SA1073	fabD	Malonyl coA-acyl carrier protein transacylase	Fatty acid biosynthesis
SA2057	fmhB	FmhB protein	Peptidoglycan biosynthesis
SA0354	rpsR	30s ribosomal protein S18	Ribosome
SA0497	rplJ	50s ribosomal protein L10	Ribosome
SAS042	rpmG	50s ribosomal protein L33 1,2,3	Ribosome
Treatment with betulinic acid and betulinaldehyde			
SA1881	kdpA	Potassium-transporting ATPase subunit A	Two-component system
SA1883	kdpE	KDP operon transcriptional regulatory protein	Two-component system
SAS042	rpmG	50s ribosomal protein L33 1,2,3	Ribosome

### Validation of Gene Expression by qPCR

**Figure 2:**
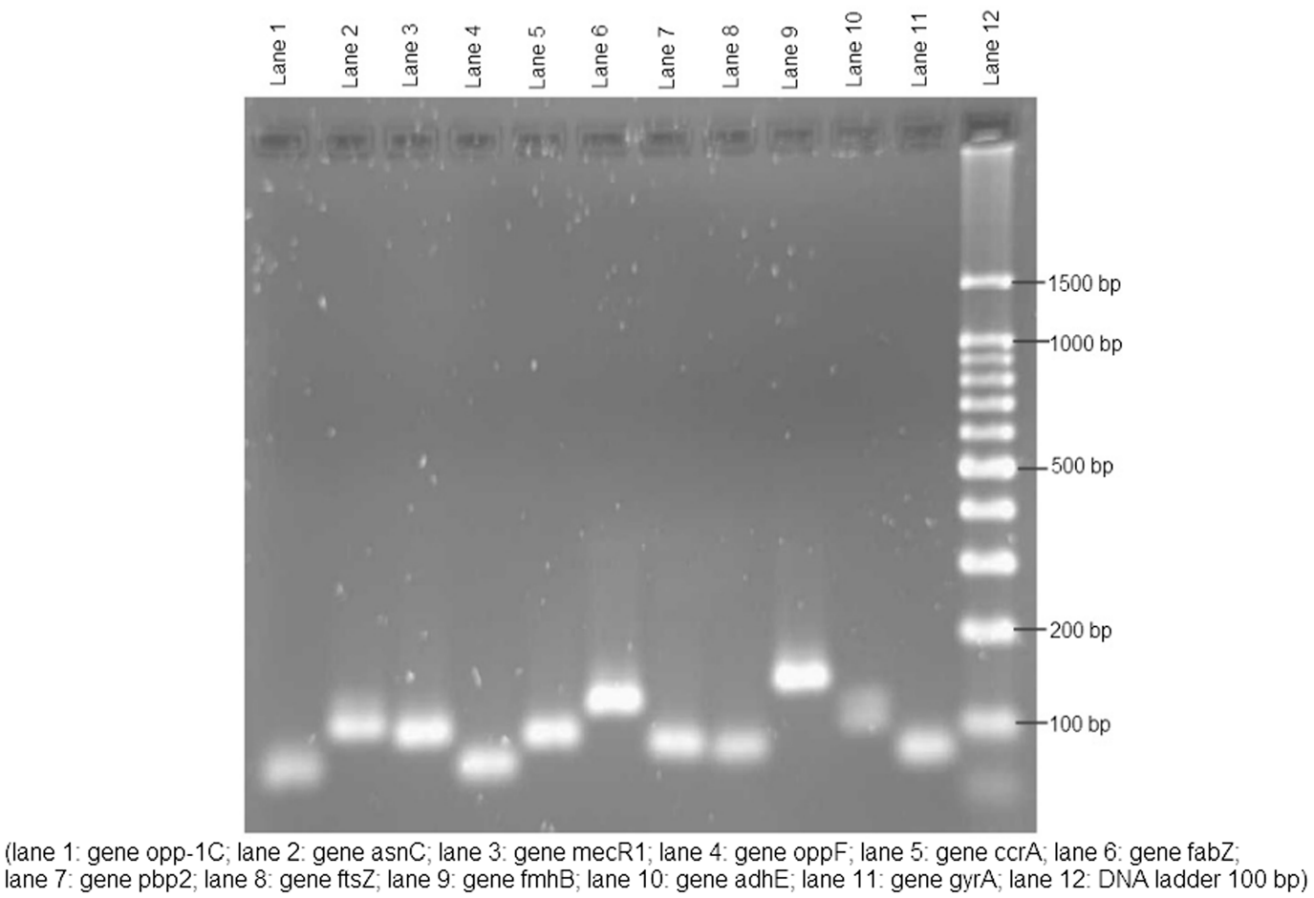
Agarose gel electrophoresis of PCR product.

The target genes selected for validation represent significant pathways in *S. aureus* identified as promising targets in the literature, and two genes selected as reference genes because they were not differentially regulated by AM, BA and BE. The target genes were *ftsZ* (cell division), *opp-1C* and *oppF* (ABC transporters), *asnC* (aminoacyl-tRNA biosynthesis), *fabZ* (fatty acid biosynthesis), *fmhB* and *pbp2* (peptidoglycan biosynthesis), *ccrA* (DNA replication), and *mecR1* (β-lactam resistance), while the reference genes were *adhE* (metabolism of fatty acid and glycolysis) and *gyrA* (DNA replication). All the target and reference genes validated showed single peaks in melting curves, indicating the amplification of specific amplicons (data not shown). The high specificity of the reactions was further confirmed by the single band and size of the amplicons observed in the agarose gel electrophoresis (Figure 2). The expression of the target genes was quantified using the relative quantification method which measures the changes in the expression of the target genes in response to the different treatment normalized to the expression of reference gene(s). The expression of genes *opp-1C*, *oppF*, *asnC* and *mecR1* was observed at greater fold-change by real-time PCR than with microarray analysis. For three other genes *fabZ*, *fmhB* and *ccrA*, the levels of gene expression did not differ markedly between the microarray data and real-time PCR analysis. Genes *ftsZ* and *pbp2* showed higher gene expression level in microarray analysis. The comparison of the gene expression level by qPCR and microarray analysis is shown in Table 8.

**Table 8:** Comparison of gene expression level by quantitative real-time PCR and microarray analysis.

N315 ORF	Gene name#	Product	Fold change	
			Real-time PCR	Microarray
SA1029	ftsZ(3)	Cell division protein	−2.2	−4.8
SA2253	opp-1C(1)	Oligopeptide transporter permease	+3.4	+2.1
SA0198	oppF(3)	Oligopeptide transport ATP-binding protein	+3.4	+2.2
SA1287	asnC(1)	Asparaginyl-tRNA synthetase	−7.4	−2.4
SA1901	fabZ(3)	(3R)-hydroxymyristoyl ACP dehydratase	−3.1	−3.7
SA2057	fmhB(1)	FmhB protein	−2.1	−2.2
SA1283	pbp2(3)	Penicillin-binding protein 2	−3.1	−4.5
SA0058	ccrA(2)	Cassette chromosome recombinase A	+2.7	+2.4
SA0039	mecR1(2)	Methicillin resistance protein	+5.9	+2.1

# Treatment of bacterial cell with pentacyclic triterpenoids:

(1) α-amyrin,

(2) betulinic acid,

(3) betulinaldehyde.

### Novel Pathways Targeted by AM, BA and BE

#### Cell division

BE inhibits genes encoding proteins FtsZ and FtsA in the cell division process of the bacterial cell. FtsZ is an essential division protein and forms a ring structure that recruits further cell division proteins. Cell division starts with the polymerization of FtsZ into the Z ring, which is the heart of the division apparatus and serves as a landing pad for the recruitment of other proteins to the division site. Suppression of FtsZ prevents cell division, leading to mass increase and eventually cell lysis. Even if inhibition of division does not result in cell death, it should prevent proliferation of the bacteria within the host and the spread of bacteria from infection sites, thus enhancing the efficiency of host-defense factors [25]. FtsA which tethers FtsZ to the membrane, recruits downstream divisome proteins and stabilizes Z rings at membrane. Thus, suppression of FtsZ and FtsA proteins inhibits growth and could exert bactericidal effect [26].

#### Two-component system

From the microarray analysis, AM, BA and BE have the potential to target the virulence factors of MRSA, which includes structures that promote adhesion, colonization and dissemination; the components responsible for cell communication and regulatory functions; the factors that facilitate evasion of the immune system; and the machineries for toxin production and excretion [27]. Expression of these factors is regulated by a range of global regulators comprising two-component regulatory systems which are sensitive to environmental signals [28], [29], [30]. The study compounds regulates three of the systems of the 16 two-component systems identified by genomic scans in *S. aureus*, i.e. Agr, Sae and Kdp.

The expression of gene *agrD* and inhibition of *agrA*, as observed in the treatment with BE, leads to the decreased production of capsule, toxins and proteases, thus decreasing the virulence of the organism. AM which inhibits *saeR* and *saeS* also decreases virulence as a result of reduction in the production in several exoproteins and cell-bound proteins involved in pathogenicity [31]. With the inhibition of *kdpA* and *kdpE* as observed in the treatment with BA and BE, the transcription of genes encoding cell wall proteins and polysaccharides, which are beneficial to colonization, are repressed [32]. As a result, the virulence of the organism is reduced with the exposure to each of the compounds.

#### ABC transporters

The up-regulation of oligopermease transporter *oppC* by AM and BA, and *oppF* by BE may increase the efficient transport of peptides for the salvage of essential amino acids from the environment of the bacterial cell. The uptake of these peptides could be to compensate for the halt in protein synthesis due to the inhibition of the aminoacyl-tRNA synthetase and ribosomes. The up-regulation of gene *sirB* as observed in the treatment with BE, indicates there could be an uptake of iron (III)-staphylobactin to satisfy its requirement for iron, which is an essential micronutrient for bacteria and serves as the catalytic center of enzymes involved in critical cellular processes such as DNA synthesis and electron transport [33]. This may represent an attempt by the organism to acquire iron for successful establishment of infection, as a response to the environmental stress caused by BE.

### Novel Targets in Existing Pathways Regulated by AM, BA and BE

#### Fatty acid biosynthesis

BE down-regulates a group of genes in the type II fatty acid synthesis (FAS) pathway, which include genes encoding for enzymes that are involved in the assembly of important cellular components in the bacteria, such as phospholipids, lipoproteins, lipopolysaccharides, myolic acids and the cell envelope [34]. The inhibition of enzymes encoded by *fabF*, *fabI*, *fabD*, *fabG* and *fabZ* may cause modification of the permeability of the phospholipid bilayer and destabilization of the cell membrane. As a result, processes such as passive transport of hydrophobic molecules, active transport of solutes and protein-protein interactions could be affected [35]. Thus, AM and BE may be able to enter the cells relatively easier via passive transport. However, due to the high partition coefficients of AM and BE at log P values of 11.01 and 9.07, respectively (Chemspider database, http://www.chemspider.com/Search.aspx), the entry of these compounds may still not increase significantly, thus the high observed MIC values at 64 and 512 µg/ml as compared to current anti-staphylococcal agents such as methicillin and vancomycin.

#### Peptidoglycan biosynthesis

Closely linked to cell division is peptidoglycan synthesis. Coordinated synthesis of the peptidoglycan is crucial for the integrity of the bacterial cell, attachment sites for virulence factors and resistance to external stress conditions [36]. BE inhibits three *mur* genes (*murA*, *murC* and *murG*), *fmhB* and *pbp2* which are involved in the synthesis initiation phase, modification, and polymerization and cross-linking of lipid II, respectively. The suppression of the synthesis of lipid II which is the substrate for Pbp2 and transpeptidation reactions results in the weakening of the peptidoglycan, leading to cell lysis and death. The majority of approved anti-staphylococcal drugs that target the peptidoglycan biosynthesis act on extracellular steps and resistance to these drugs are on the rise. An attractive alternative is to exploit the intracellular steps, involving the *mur* and *fem* genes. There are currently no known antibiotics or synthetic chemicals of therapeutic usefulness that inhibits the Mur enzymes, except for MurA which is inhibited by fosfomycin [37].

#### Aminoacyl-tRNA biosynthesis

The suppression of six aminoacyl-tRNA synthetase, namely *cysS*, *proS*, *asnC*, *thrS*, *serS* and *lysS*, by AM and BE causes the accumulation of the deacylated form of tRNA. The shortage of corresponding aminoacyl-tRNA of a required amino acid prompts the stringent response with increased levels of guanosine tetraphosphates (ppGpp) and guanosine pentaphosphates (pppGpp). RNA polymerase is inhibited, thus directly reducing the production of RNA. This leads to subsequent inhibition of all key downstream cellular processes such as the biosynthesis of protein and peptidoglycan [38]. By perturbing several metabolically important processes, the inhibition of aminoacyl-tRNA synthetase leads to the cessation of bacterial growth and the attenuation of virulence *in vivo* [39].

#### Ribosome

The assembly of the ribosome begins with the transcription of rRNA [40] and proceeds through the synthesis of ribosomal proteins [41]. In comparison to the other study compounds, BE inhibits the expression of the highest number of genes in the ribosome, with 13 and 23 of the 30S and 50S subunits, respectively. The compounds could bind to the precursor forms of rRNA and ribosomal proteins and stall the normal assembly sequence of the ribosome. As a result, continued translation could be reduced, leading to inhibition of protein synthesis and prevention of growth of the bacteria. The interference with the assembly of the ribosomal subunit from its constituent rRNA and proteins could be a new target for inhibition of antibacterial agents.

#### β-lactam resistance

MecA, the main determinant for methicillin resistance, is inducible and modulated by a signal-transduction system encoded by genes from the SCCmec [42], which comprises a sensor/signal transducer MecR1, and a transcriptional repressor, methicillin repressor MecI [43]. Treatment with BA causes the expression of gene *mecR1* that results in the production of fully functional MecR1 and MecI that appear to repress the production of pbp2a [44], [45]. The observation indicates that BA could be modified and developed into a more potent inhibitor of penicillin-binding protein (PBP) that can target MRSA. In addition, BA has the potential to restore the *in vitro* activity of β-lactams and glycopeptides against MRSA, as shown in the strong synergistic interactions with methicillin and vancomycin [17].

### Conclusions

Microbial genomics has allowed the identification of a greater number of desirable and essential antibacterial targets for further evaluation. From the transcriptional profile, AM, BA and BE regulate multiple desirable targets in essential pathways which could be further explored in the development of therapeutic agents for the treatment of *S. aureus* infections. In addition, the structure of the study compounds differ from the currently used anti-staphylococcal agents, and thus cross-resistance is unlikely and development of resistance to these novel compounds will be delayed. While the compounds have the ability to affect multiple and essential targets, the relatively high MICs in *S*. *aureus* suggests that the permeability of the compounds into the bacterial cell may not be optimum and compound modifications may be required to improve the permeability. A more detailed examination of these gene responses is also needed to further characterize the antimicrobial action of each of the regulated pathways.

## Supporting Information

Table S1 **Quantification and quality assessment of total RNA isolated from reference strain of methicillin-resistant** ***Staphylococcus aureus*** **treated with α-amyrin, betulinic acid and betulinaldehyde at ½× MIC and control.**
(DOCX)

Table S2 **Genes differentially expressed in response to α-amyrin, betulinic acid and betulinaldehyde without identified pathways.**
(DOCX)

Table S3 **Genes differentially expressed in response to α-amyrin, betulinic acid and betulinaldehyde with identified pathways.**
(DOCX)

Table S4 **Significance analysis of target and reference genes validated with quantitative real-time PCR.**
(DOCX)
