# Supplementary material for: Transcriptional Profiles of the Response of Methicillin-Resistant Staphylococcus aureus to Pentacyclic Triterpenoids
Source: PLoS One. 2013 Feb 20;8(2):e56687. doi: 10.1371/journal.pone.0056687 (PMC3577688; doi:10.1371/journal.pone.0056687)
Supplement: Table S1 — Quantification and quality assessment of total RNA isolated from reference strain of methicillin-resistant Staphylococcus aureus treated with α-amyrin, betulinic acid and betulinaldehyde at ½× MIC and control. (DOCX) [file pone.0056687.s001.docx]

**Table S1**

Quantification and quality assessment of total RNA isolated from reference strain of methicillin-resistant *Staphylococcus aureus* treated with α-amyrin, betulinic acid and betulinaldehyde at ½× MIC and control

| **Treatment** | **Replicate** | **A260/A280** | **A260/A230** | **RIN** |
| --- | --- | --- | --- | --- |
| Without treatment (control) | 1 | 1.803 | 1.935 | 8.9 |
|  | 2 | 1.814 | 1.914 | 8.6 |
|  | 3 | 1.796 | 1.885 | 8.6 |
| α-amyrin | 1 | 1.791 | 1.629 | 8.3 |
|  | 2 | 1.858 | 2.027 | 9.1 |
|  | 3 | 1.739 | 1.555 | 9.1 |
| Betulinic acid | 1 | 1.820 | 1.778 | 7.9 |
|  | 2 | 1.992 | 1.491 | 7.9 |
|  | 3 | 1.765 | 1.560 | 9.1 |
| Betulin-aldehyde | 1 | 1.847 | 1.709 | 8.0 |
|  | 2 | 1.760 | 1.505 | 8.0 |
|  | 3 | 1.915 | 2.018 | 8.0 |
