# Supplementary material for: Transcriptional Profiles of the Response of Methicillin-Resistant Staphylococcus aureus to Pentacyclic Triterpenoids
Source: PLoS One. 2013 Feb 20;8(2):e56687. doi: 10.1371/journal.pone.0056687 (PMC3577688; doi:10.1371/journal.pone.0056687)
Supplement: Table S2 — Genes differentially expressed in response to α-amyrin, betulinic acid and betulinaldehyde without identified pathways. (DOCX) [file pone.0056687.s002.docx]

**Table S2**

Genes differentially expressed in response to α-amyrin, betulinic acid and betulinaldehyde without identified pathway

| **N315 ORF** | **Gene symbol** | **Product** | **Fold change^δ^** |
| --- | --- | --- | --- |
| 1. Treatment with α-amyrin | | | |
| SA0729 | tpi | Triosephosphate isomerase | -2.1 |
| SA0996 | sdhB | Succinate dehydrogenase | -2.8 |
| SA1141 | hrcA | Heat-inducible transcriptional repressor | -2.2 |
| SA1149 | glnR | Glutamine synthetase repressor | -3.6 |
| SA1194 | msrA | Methionine sulfoxide reductase | -2.2 |
| SA1355 | nusB | N-utilization substance protein B homolog | -2.5 |
| SA2390 | panD | Aspartate 1-decarboxylase precursor | -2.2 |
| 1. Treatment with betulinic acid | | | |
| SA1113 | rbfA | Ribosome-binding factor A | -2.0 |
| SA1114 | truB | tRNA pseudouridine 5S synthase | +2.0 |
| SA2494 | cspB | Cold shock protein CspB | -2.6 |
| SA0221 | rlp | RGD-containing lipoprotein | +2.9 |
| SA1869 | sigB | RNA polymerase sigma factor | +2.0 |
| SA2455 | capC | Capsular polysaccharide synthesis enzyme Cap8C | +2.3 |
| 1. Treatment with betulinaldehyde | | | |
| SA2147 | tcaR | Transcription regulator | -4.0 |
| SA0029 | pre | Plasmid recombination enzyme | -3.6 |
| SA0996 | sdhB | Succinate dehydrogenase | -8.3 |
| SA0032 | bleO | Bleomycin resistance protein | -6.7 |
| SA1102 | frr | Ribosome-recycling factor | -7.1 |
| SA1113 | rbfA | Ribosome-binding factor A | -7.4 |
| SA1114 | truB | tRNA pseudouridine 5S synthase | +2.7 |
| SA1149 | glnR | Glutamine synthetase repressor | -6.8 |
| SA1194 | msrA | Methionine sulfoxide reductase A | -3.0 |
| SA1234 | cspA | Major cold shock protein CspA | -5.2 |
| SA1305 | hu | DNA-binding protein II | -4.1 |
| SA1355 | nusB | N-utilizing substance protein B homolog | -4.1 |
| SA0483 | clpC | Endopeptidase | -7.0 |
| SA1411 | hrcA | Heat-inducible transcriptional repressor | -4.6 |
| SA1499 | tig | Trigger factor | -5.1 |
| SA1649 | infC | Translation initiation factor IF-3 | -4.1 |
| SA1557 | ccpA | Catabolite control protein A | -3.8 |
| SA1583 | rot | Repressor of toxins | -6.0 |
| SA1629 | splC | Serine protease | +2.2 |
| SA0856 | spxA | Transcriptional regulatory response | -4.4 |
| SAS065 | hld | Delta hemolysin | -7.8 |
| SA1870 | rsbW | Serine-protein kinase RsbW | -2.4 |
| SA0219 | pflA | Formate acetyltransferase activating enzyme | -4.0 |
| SA2051 | topB | DNA topoisomerase | +2.5 |
| SA0249 | scdA | Cell division and morphogenesis-related protein | -2.6 |
| SA2336 | clpC | Dependent Clp proteinase chain | +3.2 |
| SA2455 | capC | Capsular polysaccharide biosynthesis | +2.3 |
| SA2482 | pcp | Pyrrolidone-carbohydrate peptidase | +2.3 |
| SA1835 | int | Integrase | +2.1 |
| SA0366 | ahpC | Alkyl hydroperoxidase reductase subunit C | -5.1 |
| SA0494 | nusG | Transcription antitermination protein | -2.8 |
| SA0573 | sarA | Staphylococcal accessory regulator A | -6.0 |
| SA0744 | ssp | Extracellular ECM and plasma binding protein | +3.0 |
| SA0506 | tuf | Elongation factor Tu | -4.7 |
| SA0934 | ptsH | Phosphocarrier protein Hpr | -7.6 |
| SA0148 | capE | Capsular polysaccharide synthesis enzyme | -3.1 |
| SA1098 | codY | Transcriptional repressor CodY | -6.4 |
| SA1174 | lexA | LexA repressor | -5.6 |
| SA1410 | grpE | GrpE protein (heat shock protein) | -4.9 |
| SA1382 | sodA | Superoxide dismutase SodA | -5.7 |
| SA1351 | ahrC | Arginine repressor | -2.8 |
| SA1871 | rsbV | Anti-sigmaB factor antagonist | -4.5 |
| SA1872 | rsbU | Sigma B regulation protein | -2.3 |
