# Supplementary material for: Transcriptional Profiles of the Response of Methicillin-Resistant Staphylococcus aureus to Pentacyclic Triterpenoids
Source: PLoS One. 2013 Feb 20;8(2):e56687. doi: 10.1371/journal.pone.0056687 (PMC3577688; doi:10.1371/journal.pone.0056687)
Supplement: Table S3 — Genes differentially expressed in response to α-amyrin, betulinic acid and betulinaldehyde with identified pathways. (DOCX) [file pone.0056687.s003.docx]

**Table S3**

Genes differentially expressed in response to α-amyrin, betulinic acid and betulinaldehyde with identified pathway

| **N315 ORF** | **Gene symbol** | **Product** | **Fold change^δ^** | **Pathway** |
| --- | --- | --- | --- | --- |
| 1. Treatment with α-amyrin | | | | |
| SA1117 | pnpA | Polyribonucleotide nucleotidyltransferase | -2.2 | Purine and pyrimidine metabolism |
| SA1150 | glnA | Glutamine ammonia ligase | -2.4 | Metabolic pathway; nitrogen metabolism |
| SA1533 | ackA | Acetate/propionate kinase | -2.2 | Pyruvate metabolism |
| SA1817 | sec3 | Enterotoxin type C-3 | -2.1 | *Staphylococcus aureus* infection |
| SA1963 | mtlD | Mannitol-1-phosphate-5-dehydrogenase | -2.7 | Fructose and mannose metabolism |
| SA2207 | hlgA | Gamma-hemolysin chain II precursor | -3.3 | *Staphylococcus aureus* infection |
| 1. Treatment with betulinic acid | | | | |
| SA1819 | tst | Toxic shock syndrome toxin I | -2.0 | *Staphylococcus aureus* infection |
| SA1070 | recG | ATP-dependent DNA helicase | +2.0 | Homologous recombinant |
| SA1225 | lysC | Aspartokinase | +2.1 | Metabolic pathway; lysine biosynthesis |
| SA1858 | ilvD | Dihydroxy-acid dehydratase | +2.6 | Metabolic pathway; valine, leucine and isoleucine biosynthesis |
| SA1863 | leuB | 3-isopropyl malate dehydrogenase | +2.2 | Metabolic pathway; valine, leucine and isoleucine biosynthesis |
| SA2122 | hutU | Urocanate hydratase | +2.0 | Metabolic pathway; histidine metabolism |
| SA2207 | hlgA | Gamma-hemolysin chain II precursor | -2.5 | *Staphylococcus aureus* infection |
| SA2214 | bioA | Adenosylmethionine-8-amino-7-oxononoate aminotransferase | +2.5 | Metabolic pathway; biotin metabolism |
| SA0430 | gltB | Glutamate synthase large sububit | +2.2 | Metabolic pathway; alanine, aspartate and glutamine metabolism |
| SA0431 | gltD | NADH-glutamate synthase small subunit | +2.7 | Metabolic pathway; alanine, aspartate and glutamine metabolism |
| 1. Treatment with betulinaldehdye | | | | |
| SA0309 | geh | Glycerol ester hydrolase | -6.0 | Metabolic pathway; glycerolipid metabolism |
| SA0419 | metB | Cystathionine gamma synthase | +3.8 | Metabolic pathway; cysteine and methionine metabolism |
| SA0107 | spa | Immunoglobulin G binding protein A precursor | -3.5 | *Staphylococcus aureus* infection |
| SA1052 | gmk | Guanylate kinase | -4.3 | Metabolic pathway; purine metabolism |
| SA1072 | plsX | Fatty acid/phospholipid synthesis protein | -3.3 | Glycerophospholipid metabolism |
| SA1088 | sucC | Succinyl-coA ligase subunit beta | -3.7 | Metabolic pathway; citrate cycle |
| SA1089 | sucD | Succinyl-coA ligase subunit alpha | -7.1 | Metabolic pathway; citrate cycle |
| SA1142 | glpD | Aerobic glycerol-3-phosphate dehydrogenase | -3.5 | Glycerophospholipid metabolism |
| SA1150 | glnA | Glutamine ammonia ligase | -3.1 | Metabolic pathway; nitrogen metabolism |
| SA1770 | katA | Catalase | -2.1 | Metabolic pathway; tryptophan metabolism |
| SA1177 | tkt | Transketolase | -4.1 | Metabolic pathway; pentose phosphate pathway |
| SA1200 | trpG | Anthranilase synthase component II | +2.8 | Metabolic pathway; phenylalanine, tyrosine and tryptophan biosynthesis |
| SA1226 | asd | Aspartate semialdehyde dehydrogenase | +2.1 | Metabolic pathway; glycine, threonine and serine metabolism |
| SA1244 | odhB | Dihydrolipoamide acetyltransferase | -5.1 | Metabolic pathway; citrate cycle |
| SA0134 | drm | Phosphopentamutase | -3.1 | Pentose phosphate pathway; purine metabolism |
| SA1299 | aroC | Chorismate synthase | +2.6 | Metabolic pathway; phenylalanine, tyrosine and tryptophan biosynthesis |
| SA1461 | apt | Adenine phosphoribosyltransferase | -2.5 | Metabolic pathway; purine metabolism |
| SA1487 | folC | Folylpolyglutamine synthase | -3.1 | Metabolic pathway; folate biosynthesis |
| SA1496 | hemA | Glutamyl-tRNA reductase | -4.0 | Metabolic pathways; porphyrin and chlorophyll metabolism |
| SA1517 | citC | Isocitrate dehydrogenase | -2.3 | Metabolic pathway; citrate cycle (TCA) |
| SA1520 | pykA | Pyruvate kinase | -5.3 | Metabolic pathway; glycolysis/gluconeogenesis; pyruvate metabolism |
| SA0008 | hutH | Histidine ammonia lyase | -2.0 | Metabolic pathway; histidine metabolism |
| SA1521 | pfkA | 6-phosphofructokinase | -3.5 | Metabolic pathway; glycolysis/gluconeogenesis; pentose phosphate pathway |
| SA1522 | accA | Acetyl-coA-carboxylase alpha subunit | -2.5 | Metabolic pathway; fatty acid biosynthesis; pyruvate metabolism |
| SA1525 | dnaE | DNA polymerase III alpha chain | +2.1 | Metabolic pathway; DNA replication; homologous recombination |
| SA1531 | ald | Alanine dehydrgenase | -4.2 | Metabolic pathway; alanine, aspartate and glutamate metabolism |
| SA1150 | ackA | Acetate/propionate kinase | -2.3 | Metabolic pathway; pyruvate metabolism; propanoate metabolism |
| SA1609 | pckA | Phosphoenoylpyruvate carboxykinase | -2.4 | Metabolic pathway; glycolysis/gluconeogenesis; citrate cycle (TCA) |
| SA0177 | argJ | Arginine biosynthesis bifunctional protein | +3.8 | Metabolic pathway; arginine and proline metabolism |
| SA0178 | argC | N-acetyl-gamma-glutamyl phosphate reductase | +2.9 | Metabolic pathway; arginine and proline metabolism |
| SA1817 | sec3 | Enterotoxin type C-3 | -22.8 | *Staphylococcus aureus* infection |
| SA1858 | ilvD | Dihydroxy-acid dehydrogenase | +4.5 | Metabolic pathway; valine, leucine and isoleucine biosynthesis |
| SA1859 | ilvB | Acetolactate synthase large subunit | +3.3 | Metabolic pathway; valine, leucine and isoleucine biosynthesis |
| SA1862 | leuA | 2-isopropylmalate synthase | +3.2 | Metabolic pathways; valine, leucine and isoleucine biosynthesis; pyruvate metabolism |
| SA1864 | leuC | Isopropylmalate isomerase large subunit | +2.7 | Metabolic pathways; valine, leucine and isoleucine biosynthesis; C5-branched dibasic acid metabolism |
| SA1905 | atpD | ATP synthase subunit B | -2.7 | Metabolic pathway; oxidative phosphorylation |
| SA1906 | atpG | ATP synthase gamma chain | -4.9 | Metabolic pathway; oxidative phosphorylation |
| SA1908 | atpH | ATP synthase delta chain | -6.3 | Metabolic pathway; oxidative phosphorylation |
| SA1909 | atpF | ATP synthase B chain | -4.2 | Metabolic pathway; oxidative phosphorylation |
| SA1910 | atpE | ATP synthase subunit C | -5.6 | Metabolic pathway; oxidative phosphorylation |
| SA1911 | atpB | ATP synthase subunit A | -3.1 | Metabolic pathway; oxidative phosphorylation |
| SA1914 | upp | Uracil phosphoribosyl transferase | -3.7 | Metabolic pathway; pyrimidine metabolism |
| SA1918 | pdp | Pyrimidine-nucleoside phosphorylase | -3.7 | Metabolic pathway; pyrimidine metabolism |
| SA1940 | deoD | Purine nucleoside phosphorylase | -2.5 | Metabolic pathway; purine metabolism; pyrimidine metabolism |
| SA1962 | mtlA | PTS system, mannitol specific IIA component | -3.5 | Fructose and mannose metabolism; phosphotransferase system (PTS) |
| SA1991 | lacG | 6-phospho-beta-galactosidase | -6.0 | Galactose metabolism |
| SA1993 | lacF | PTS system, lactose specific IIA component | -4.2 | Galactose metabolism; phosphotransferase system |
| SA1994 | lacD | Tagatose-1,6-diphosphate aldolase | -2.7 | Galactose metabolism |
| SA1995 | lacC | Tagatose phosphate kinase | -2.5 | Galactose metabolism |
| SA1996 | lacB | Galactose-6-phosphate isomerase | -4.9 | Galactose metabolism |
| SA1997 | lacA | Galactose-6-phosphate isomerase | -7.6 | Galactose metabolism |
| SA1998 | lacR | Lactose phosphotransferase repressor | -3.5 | Galactose metabolism |
| SA2023 | rpoA | DNA-directed RNA polymerase alpha subunit | -3.7 | Metabolic pathway; purine metabolism; pyrimidine metabolism |
| SA2084 | ureC | Urease subunit alpha | +4.3 | Metabolic pathway; arginine and proline metabolism |
| SA0898 | menB | Naphthoate synthase | -3.5 | Metabolic pathway; ubiquinone and other terpenoid-quinone biosynthesis |
| SA0727 | sbi | IgG-binding protein SBI (pathogenesis) | -6.5 | *Staphylococcus aureus* infection |
| SA2207 | hlgA | Gamma-hemolysin chain II precursor | -6.2 | *Staphylococcus aureus* infection |
| SA2294 | gntK | Gluconokinase | +2.9 | Metabolic pathway; pentose phosphate pathway |
| SA2326 | ptsG | PTS system, glucose-specific IIABC component | +2.6 | Amino sugar and nucleotide sugar metabolism; Phosphotransferase system (PTS) |
| SA0911 | qoxC | Quinol oxidase polypeptide III QoxC | -6.3 | Metabolic pathway; oxidative phosphorylation |
| SA0912 | qoxB | Quinol oxidase polypeptide I | -2.8 | Metabolic pathway; oxidative phosphorylation |
| SA2348 | crtN | Squalene desaturase | +2.2 | Carotenoid biosynthesis |
| SA2391 | panC | Pantothenate synthetase | -2.9 | Metabolic pathway; beta-alanine metabolism |
| SA2390 | panD | Aspartate 1-decarboxylase precursor | -5.5 | Metabolic pathway; beta-alanine metabolism |
| SA2392 | panB | 3-methyl-2-oxobutanoate hydroxymethyltransferase | -3.4 | Metabolic pathway; beta-alanine metabolism |
| SA0915 | folD | FolD bifunctional protein | -5.0 | One carbon pool by folate |
| SA2427 | arcB | Ornithine carbomoyltransferase | -2.1 | Metabolic pathways; arginine and proline metabolism |
| SA2463 | lip | Triacylglycerol lipase precursor | -2.4 | Metabolic pathway; glycerolipid metabolism |
| SA0309 | geh | Glycerol ester hydrolase | -6.1 | Metabolic pathway; glycerolipid metabolism |
| SA0353 | ssb | Single-stranded DNA-binding protein | -5.3 | DNA replication; mismatch repair |
| SA0935 | ptsI | Phosphoenolpyruvate-protein phosphotransferase | -5.2 | Phosphotransferase system |
| SA0430 | gltB | Glutamate synthase large subunit | +3.7 | Metabolic pathway; alanine, aspartate and glutamate metabolism; nitrogen metabolism |
| SA0431 | gltD | NADH-glutamate synthase small subunit | +3.3 | Metabolic pathway; alanine, aspartate and glutamate metabolism; nitrogen metabolism |
| SA0501 | rpoC | DNA-directed RNA polymerase beta subunit | -3.1 | Metabolic pathway; purine metabolism; pyrimidine metabolism |
| SA0534 | vraB | Acetyl-coA acetyltransferase | +3.6 | Metabolic pathway; *multiple metabolism*; two component system |
| SA0538 | ung | Uracil-DNA glycosylase | +2.6 | Base excision repair |
| SA0548 | mvaD | Mevalonate diphosphate decarboxylase | +2.0 | Terpenoid backbone biosynthesis |
| SA0946 | pdhD | Dihydrolipoamide dehydrogenase | -8.0 | Metabolic pathway; *multiple metabolism* |
| SA0562 | adh1 | Alcohol dehydrogenase | -4.2 | Metabolic pathway; *multiple metabolism* |
| SA0719 | trxB | Thioredoxin-disulfide reductase | -2.9 | Pyrimidine metabolism |
| SA0728 | pgk | Phosphoglycerate kinase | -5.2 | Metabolic pathway; glycolysis/gluconeogenesis |
| SA0729 | tpiA | Triosephosphate isomerase | -8.3 | Metabolic pathway; glycolysis/gluconeogenesis; fructose and mannose metabolism |
| SA0730 | pgm | Phosphoglyceromutase | -4.6 | Metabolic pathway; glycolysis/gluconeogenesis; glycine, serine and threonine metabolism |
| SA0510 | sdrD | Cell adhesion | -2.5 | *Staphylococcus aureus* infection |
| SA0965 | ctaB | Protoheme IX farnesyltransferase | -2.3 | Metabolic pathway; oxidative phosphorylation |
| SA0823 | pgi | Glucose-6-phosphate isomerase | -4.9 | Metabolic pathway; glycolysis/gluconeogenesis; pentose phosphate pathway |
| SA0597 | tagD | Teichoic acid biosynthesis protein D | -2.1 | Glycerophospholipid metabolism |
| SA0727 | gap | Glyceraldehyde-3-phosphate dehydrogenase | -5.5 | Metabolic pathway; glycolysis/gluconeogenesis |
| SA0819 | gudB | NAD-specific glutamase dehydrogenase | -4.1 | Metabolic pathway; alanine, aspartate and glutamine metabolism |
| SA0458 | prs | Ribose-phosphate pyrophosphokinase | -3.3 | Metabolic pathway; pentose phosphate pathway; purine metabolism |
| SA0375 | guaB | Inositol-monophosphate dehydrogenase | -3.0 | Metabolic pathway; purine metabolism |
| SA0376 | guaA | GMP synthase | -3.8 | Metabolic pathway; purine metabolism |
| SA0218 | pflB | Formate acetyltransferase | -3.3 | Metabolic pathway; pyruvate metabolism |
| SA2341 | rocA | 1-pyrroline-5-carboxylate dehydrogenase | -3.7 | Metabolic pathway; alanine, aspartate and glutamate metabolism |
| SA2405 | betA | Choline dehydrogenase | +2.2 | Glycine, serine and threonine metabolism |
| SA0122 | butA | Acetoin reductase | -3.4 | Butanoate metabolism |
| SA1439 | udk | Uridine kinase | -3.8 | Metabolic pathway; pyrimidine metabolism |
| SA1101 | smbA/ pyrH | Uridylate kinase | -2.2 | Metabolic pathway; pyrimidine metabolism |
| SA1377 | glcK | Glucokinase | -2.3 | Metabolic pathway; glycolysis/gluconeogenesis; galactose metabolism |
| SA0995 | sdhA | Succinate dehydrogenase | -3.9 | Metabolic pathway; citrate cycle; oxidative phosphorylation |
| SA0996 | sdhB | Succinate dehydrogenase | -5.2 | Metabolic pathway; citrate cycle; oxidative phosphorylation |
| SA1342 | gnd | 6-phosphogluconate dehydrogenase | -4.4 | Metabolic pathway; pentose phosphate pathway |
| SA1259 | dfrA | Dihydrofolate reductase | -2.7 | Metabolic pathway; folate biosynthesis |
| SA1965 | glmM | Phosphoglucosamine mutase GlmM | -2.8 | Metabolic pathway; amino sugar and nucleotide sugar metabolism |
| SA1927 | fbaA | Fructose bisphosphate aldolase | -4.9 | Metabolic pathway; glycolysis/gluconeogenesis; pentose phosphate pathway |
