# Supplementary material for: Transcriptional Profiles of the Response of Methicillin-Resistant Staphylococcus aureus to Pentacyclic Triterpenoids
Source: PLoS One. 2013 Feb 20;8(2):e56687. doi: 10.1371/journal.pone.0056687 (PMC3577688; doi:10.1371/journal.pone.0056687)
Supplement: Table S4 — Significance analysis of target and reference genes validated with quantitative real-time PCR. (DOCX) [file pone.0056687.s004.docx]

**Table S4**

Significance analysis of target and reference genes validated with quantitative real-time PCR

| **Treatment** | **Gene** | **C_t_ _(test)_ + SD** | **C_t_ _(control)_ + SD** | **P value** | **Significance** |
| --- | --- | --- | --- | --- | --- |
| α-amyrin | fmhB | 26.6944 + 0.1338 | 25.6300 + 0.1645 | 0.0001 | Yes |
|  | opp-1C | 23.4789 + 0.3846 | 25.2556 + 0.3213 | 0.0001 | Yes |
|  | asnC | 28.2256 + 0.2710 | 25.3433 + 0.2988 | 0.0001 | Yes |
|  | adhE | 24.6733 + 0.3608 | 24.7011 + 0.2405 | 0.8258 | No |
|  | gyrA | 24.7289 + 0.2962 | 24.7067 + 0.3759 | 0.8961 | No |
| Betulinic acid | mecR1 | 22.8467 + 0.1792 | 25.4211 + 0.3742 | 0.0001 | Yes |
|  | oppF | 23.4444 + 0.2527 | 25.1467 + 0.2185 | 0.0001 | Yes |
|  | ccrA | 23.1533 + 0.2409 | 24.5378 + 0.1992 | 0.0001 | Yes |
|  | adhE | 24.5533 + 0.3189 | 24.6089 + 0.3322 | 0.7294 | No |
|  | gyrA | 24.6067 + 0.3497 | 24.4356 + 0.2040 | 0.3183 | No |
| Betulinaldehyde | fabZ | 23.3544 +0.3255 | 21.7122 + 0.1902 | 0.0016 | Yes |
|  | pbp2 | 24.1967 + 0.1185 | 22.6378 + 0.2572 | 0.0001 | Yes |
|  | ftsZ | 22.3333 + 0.3695 | 21.0889 + 0.2031 | 0.0001 | Yes |
|  | adhE | 24.3378 + 0.2688 | 24.2200 + 0.2616 | 0.3578 | No |
|  | gyrA | 24.2100 + 0.3083 | 24.2586 + 0.2176 | 0.7434 | No |
